# The P body protein LSm1 contributes to stimulation of hepatitis C virus translation, but not replication, by microRNA-122

**DOI:** 10.1093/nar/gkt941

**Published:** 2013-10-18

**Authors:** Ashley P. E. Roberts, Rachel Doidge, Alexander W. Tarr, Catherine L. Jopling

**Affiliations:** School of Pharmacy, Centre for Biomolecular Sciences, University of Nottingham, University Park, Nottingham NG7 2RD, UK

## Abstract

The P body protein LSm1 stimulates translation and replication of hepatitis C virus (HCV). As the liver-specific microRNA-122 (miR-122) is required for HCV replication and is associated with P bodies, we investigated whether regulation of HCV by LSm1 involves miR-122. Here, we demonstrate that LSm1 contributes to activation of HCV internal ribosome entry site (IRES)-driven translation by miR-122. This role for LSm1 is specialized for miR-122 translation activation, as LSm1 depletion does not affect the repressive function of miR-122 at 3′ untranslated region (UTR) sites, or miR-122–mediated cleavage at a perfectly complementary site. We find that LSm1 does not influence recruitment of the microRNA (miRNA)-induced silencing complex to the HCV 5′UTR, implying that it regulates miR-122 function subsequent to target binding. In contrast to the interplay between miR-122 and LSm1 in translation, we find that LSm1 is not required for miR-122 to stimulate HCV replication, suggesting that miR-122 regulation of HCV translation and replication have different requirements. For the first time, we have identified a protein factor that specifically contributes to activation of HCV IRES-driven translation by miR-122, but not to other activities of the miRNA. Our results enhance understanding of the mechanisms by which miR-122 and LSm1 regulate HCV.

## INTRODUCTION

Hepatitis C virus (HCV) is a major global cause of disease, with 2–3% of the population infected. The virus causes chronic liver infections that may progress to cirrhosis and hepatocellular carcinoma. The current drug regime is poorly tolerated and frequently ineffective, and there is an urgent need for better understanding of HCV biology that may lead to novel therapeutic strategies (1). HCV is a positive sense single-stranded RNA virus of the *Flaviviridae* family. The HCV genome is ∼9.6 kb in length, with a single open reading frame encoding structural (core, E1, E2 and p7) and nonstructural (NS2, NS3, NS4A, NS4B, NS5A and NS5B) proteins flanked by highly structured 5′ and 3′ UTRs (2). Both UTRs are required for viral RNA replication, while the 5′ UTR contains an internal ribosome entry site (IRES) that directly recruits the 40S ribosomal subunit and eukaryotic initiation factor 3 to initiate cap-independent translation of the viral polyprotein. In the cytoplasm, the viral RNA first serves as a template for translation before replication takes place in association with endoplasmic reticulum–derived membranes, with the viral NS5B RNA-dependent RNA polymerase mediating synthesis of new − and + strand HCV RNA.

The highly expressed liver-specific microRNA-122 (miR-122) is essential for HCV replication and is a promising antiviral target (3,4). Excitingly, an oligonucleotide miR-122 inhibitor, miravirsen, recently completed phase 2a clinical trials in HCV-infected patients. The drug resulted in prolonged dose-dependent reduction in viral RNA with minimal adverse effects and without evidence of viral resistance (5), emphasizing the clinical importance of HCV regulation by miR-122 and the need for better understanding of its mechanism. MicroRNAs (miRNAs) are small (21–23 nt) noncoding RNA molecules that canonically function by binding to partially complementary sites in the 3′UTR of mRNA targets, leading to translational repression and mRNA degradation (6). In contrast, miR-122 regulates HCV by interacting with two adjacent sites in the viral 5′UTR, immediately upstream of the IRES, and positively regulating the viral replication cycle (3,7).

The mechanism by which miR-122 regulates HCV is not fully understood. miR-122 does not directly affect HCV RNA synthesis in cells or in isolated replication complexes (8,9). Previous work from our group and others has shown that miR-122 binding to the HCV 5′UTR stimulates HCV IRES-driven translation (10–13). However, this minor effect is not sufficient to account for the major role miR-122 plays in the HCV replication cycle, as miR-122 binding site mutation leads to a more severe replication defect than IRES mutations that repress translation to a similar extent (14). miR-122 binding was recently shown to stabilize HCV RNA by protection from degradation by the 5′-3′ exonuclease Xrn1, and it was proposed that previous observations of activation of translation by miR-122 could instead be explained by RNA stabilization (15). However, protection from degradation is also insufficient to explain the essential role for miR-122 in HCV replication, as HCV RNA with mutated miR-122 binding sites does not replicate even when Xrn1 is depleted (15). Moreover, miR-122 with mutations that abolish regulation of HCV replication can still protect HCV RNA from degradation by Xrn1 (16), and mutation of miR-122 binding sites does not affect Xrn1 binding to the HCV 5′UTR (13). Instead, it appears that miR-122 must promote another stage of the HCV replication cycle, in addition to translation and/or RNA stability, by an as yet undetermined mechanism.

miRNAs bind to 3′UTR sites and mediate their repressive function in association with a complex of proteins known as the miRNA-induced silencing complex (miRISC), which has essential Argonaute (Ago1–4 in mammals) and GW182 (TNRC6A-C in mammals) components (6). The Argonaute proteins are required for miR-122 to regulate HCV (11,12), but it is not yet known whether additional miRISC proteins or cofactors are necessary. Interestingly, processing (P) bodies are associated with both HCV replication and miRNA function (17,18), raising the possibility that P bodies may be involved in miR-122–mediated regulation of HCV. P bodies are cytoplasmic foci where translationally repressed mRNAs accumulate and are degraded (19). P bodies may also function as sites of mRNA storage before a return to active translation (18), although the extent to which this occurs has recently been called into question (20). miRNAs, miRNA-repressed mRNAs and protein components of the miRNA repression machinery associate with P bodies, suggesting that these foci may be important for miRNA function (18,21). P body proteins are implicated in the replication of several RNA viruses, including brome mosaic virus, West Nile virus, poliovirus and HCV (17,22–24). The P body proteins Rck/p54 (DDX6), PatL1 and LSm1 all contribute to HCV replication (17,25), while Xrn1 represses replication in one study (15) but shows no effect in another (17). HCV replication leads to relocalization of Rck/p54, PatL1, LSm1 and Xrn1, but not Dcp2, from P bodies to lipid droplets, where they colocalize with the HCV Core protein (26–28). This suggests that these proteins are recruited from the P bodies to sites of HCV replication.

As miR-122 is present in P bodies (18), we considered the possibility that regulation of HCV by miR-122 and P body proteins might involve a common mechanism. While Rck/p54 regulation of HCV is independent of miR-122 (25,29), and miR-122 may protect HCV RNA from degradation by Xrn1 (15), the interplay between other P body components and miR-122 in HCV regulation has not been examined. We chose to focus on the role of LSm1 in miR-122–mediated regulation of HCV. In eukaryotes, LSm proteins form two highly conserved heteroheptameric ring structures: the LSm2–8 complex, involved in nuclear RNA processing, and the LSm1–7 complex, which is located in P bodies (30,31). In yeast, the LSm1–7 complex associates with deadenylated RNA and several RNA decay factors including Pat1, protecting mRNAs from 3′ end trimming but promoting decapping and subsequent 5′-3′ RNA degradation (30,32,33). Purified LSm1–7-Pat1 binds directly to mRNA near the 3′ end, with higher affinity for oligoadenylated RNA (34). Intriguingly, LSm proteins are homologous to the bacterial Hfq protein, which has similar functions in mRNA decay and bacteriophage Qβ replication. Hfq also acts as a chaperone to mediate regulation of gene expression by small RNAs (35), raising the possibility that LSm proteins may have similar roles in small RNA activity in eukaryotes.

In this study, we assess the role for LSm1 in miR-122–mediated regulation of HCV. We demonstrate that LSm1 contributes to miR-122–mediated activation of HCV IRES-driven translation in both luciferase reporters and infectious bicistronic HCV. LSm1 depletion does not affect miR-122–mediated repression via 3′UTR sites, indicating a specialized role for this protein in regulating miR-122 activity at the HCV 5′UTR. We show that LSm1 depletion does not affect the association between miR-122-RISC and HCV RNA, implying that it functions to regulate miR-122 activity subsequent to target binding. Finally, we find that LSm1 is not required for miR-122 to regulate HCV replication, suggesting that miR-122–mediated regulation of HCV translation and replication are distinct processes with different host factor requirements.

Taken together, we have identified LSm1 as a protein that contributes specifically to miR-122–mediated stimulation of translation from the HCV IRES, but not to other repressive functions of miR-122 or to the role of the miRNA in HCV replication. Our results provide new insight into the interplay between miRNAs, P bodies and viral replication with potential relevance to future antiviral drug development.

## MATERIALS AND METHODS

### Plasmids, *in vitro* transcription and RNA oligonucleotides

The plasmids pLUC122si, pLUC122x2 and wild-type and mutant forms of p5′LUC3′ have been described previously (3,11,36). pH77ΔE1/p7 was a kind gift of Stanley Lemon (37). pH77ΔE1/p7-AAG was generated by replacing the wild-type NS5B coding sequence with a GDD-AAG active site mutant from a full-length H77 clone, also a gift of Stanley Lemon (38). The double miR-122 binding site mutant pH77ΔE1/p7-S1+2:p3+4 was described previously (36). Two plasmids encoding infectious HCV RNAs, pBi-Gluc-H77C(1a)/JFH and pFL-J6/JFH1, were kind gifts of Charles Rice (39). *In vitro* transcription was carried out using the T7 Megascript kit (Ambion) according to the manufacturer’s instructions with *EcoRI*-linearized p5′LUC3′ or *XbaI*-linearized pH77ΔE1/p7, pBi-Gluc-H77C(1a)/JFH1 or pFL-J6/JFH1 as templates. The capped polyadenylated *Renilla* luciferase transfection control RNA was synthesized from a linearized pSV40-RL (Promega) template using the mMessage mMachine kit (Ambion) and polyadenylated using the Poly(A) tailing kit (Ambion). miRNA duplexes and 2′-O-methylated oligonucleotides have been described previously (36). Synthetic pre-miR-122 had the sequence 5′-UGGAGUGUGACAAUGGUGUUUGUGUCUAAACUAUCAAACGCCAUUAUCACACUAAAUA-3′ and was purchased from Dharmacon.

### Cell culture and transfection

Huh7 and Huh7.5 cells were cultured as previously described (11). siRNAs are shown in Supplementary Table S1 and were delivered into cells at 20 nM final concentration using Lipofectamine RNAiMax (Invitrogen). Cells were cultured for 48 h before RNA or DNA transfection. For luciferase experiments, cells were transfected with 0.2 µg of 5′LUC3′ RNA (or mutant variants) with 0.01 µg capped polyadenylated *Renilla* luciferase RNA, or 0.5 µg of firefly luciferase plasmid DNA with 0.05 µg of pSV40-RL. Twenty nanomolar randomized or miR-122–specific 2′O-methylated oligonucleotide, miR122wt duplex or miR-122p3+4 mutant duplex was also included in the transfections, which were performed using Lipofectamine 2000 (Invitrogen) as in (11). Cells were harvested at 6 h after transfection for RNA or 24 h for DNA transfections. Cells cultured in 24-well plates were harvested in Passive Lysis Buffer (Promega) and luciferase activity measured with the Dual luciferase assay system (Promega) using a Glomax luminometer (Promega). To quantify luciferase RNA, cells were cultured in six-well plates and TRI reagent extraction and quantitative reverse transcriptase-polymerase chain reaction (qPCR) carried out as described below.

Electroporation was used to introduce wild-type or mutant H77ΔE1/p7, Bi-Gluc-H77C(1a)/JFH1 or FL-J6/JFH1 RNA into Huh 7 or Huh7.5 cells, and to transfect Huh7 cells with 5′LUC3′ RNA for immunoprecipitation experiments. Electroporation was carried out using the Neon system (Invitrogen) according to the manufacturer’s instructions. Where included, siRNA or 2′-O-methylated oligonucleotide treatment was for 48 h before electroporation. For HCV replication experiments, two siRNA transfections were conducted at 72 and 24 h before electroporation to ensure that knockdown was maintained in cells harvested at 24 h after electroporation. Cells (4 × 10^5^) were resuspended in 10 μl of buffer R and mixed with 1 μg of wild-type or mutant H77ΔE1/p7, Bi-Gluc-H77C(1a)/JFH1, FL-J6/JFH1 or 5′LUC3′ RNA, and 20 pmoles 122-2′Ome or pre-miR-122 where included, before electroporation with a single pulse at 1300 V for 30 ms. For immunoprecipitation experiments, three electroporations were pooled and plated on a 10-cm plate. Cells electroporated with HCV RNA were plated in six-well plates and total RNA extracted at 6 and 24 h after electroporation using TRI reagent. A fraction of cells electroporated with Bi-Gluc-H77C(1a)/JFH1 RNA were plated in 24-well plates for luciferase assays. Ten microliters of cell supernatant from triplicate wells was harvested at 1, 2, 3, 4, 6 and 24 h time points in luciferase lysis buffer (NEB), and assayed with *Gaussia* luciferase assay reagent (NEB).

### Immunoprecipitation

Immunoprecipitation of Argonaute complexes was performed on electroporated cells cultured in 10-cm plates as described in (40), except that the monoclonal antibody 11A9 (Sigma SAB4200085) was used to immunoprecipitate Ago2. RNA was isolated from 10% of the input cell lysate, the Ago2 immunoprecipitate and a normal rat IgG control immunoprecipitate.

### RNA isolation, northern blotting and qPCR

RNA was extracted using TRI reagent (Sigma) according to the manufacturer’s protocol. Northern blot analysis of HCV and γ-actin RNA was carried out as described previously (36). qPCR was performed using GoTaq qPCR Master Mix (Promega), following reverse transcription using Superscript III and random primers, as described in (11). qPCR primer sequences are shown in Supplementary Table S2. The primer pair HCV qF and HCV qR bind to the HCV 5′UTR and were used to amplify both H77ΔE1/p7 and 5′LUC3′ RNA. JFH1 qF and JFH1 qR primers were used to amplify Bi-Gluc-H77C(1a)/JFH1 and FL-J6/JFH1 RNA. Primer specificity was confirmed by agarose gel electrophoresis of PCR products and dissociation curves. qPCR to detect miR-122 and U6 small nuclear RNA (snRNA) was carried out using specific miRNA Taqman assay kits (Applied Biosystems) according to the manufacturer’s protocol. qPCR was carried out using a Stratagene Mx3005P machine, and data were analyzed by the 2^-ΔΔCt^ method relative to the actin mRNA or U6 control for total RNA experiments, or fitted to a standard curve of *in vitro* transcribed 5′LUC3′ or H77ΔE1/p7 RNA using the MxPro software for immunoprecipitation experiments.

### Western blotting

Protein samples were obtained by resuspension of cell pellets in 1× sodium dodecyl sulphate-polyacrylamide gel electrophoresis (SDS-PAGE) loading dye, and separated by electrophoresis on 15% SDS-PAGE gels before semidry transfer to polyvinylidene difluoride (PVDF) membrane. LSm1 was detected using the antibody GW22100F (Sigma) and β-tubulin using the antibody ab6046 (Abcam).

### Statistical analysis

All data represent averages of at least three independent biological replicates, with error bars representing standard deviation. Statistical analysis was carried out by two-tailed Student’s *t*-test for unpaired samples of equal variance. *P < 0.05, **P < 0.005, ***P < 0.0005.

## RESULTS

### LSm1 contributes to miR-122-dependent stimulation of translation from the HCV IRES

miR-122 and LSm1 both contribute to HCV replication, at least in part by stimulating HCV translation (11,17), and both are localized to P bodies (18). This led us to investigate the possibility that these host factors might cooperate to regulate HCV.

**Figure 1.**
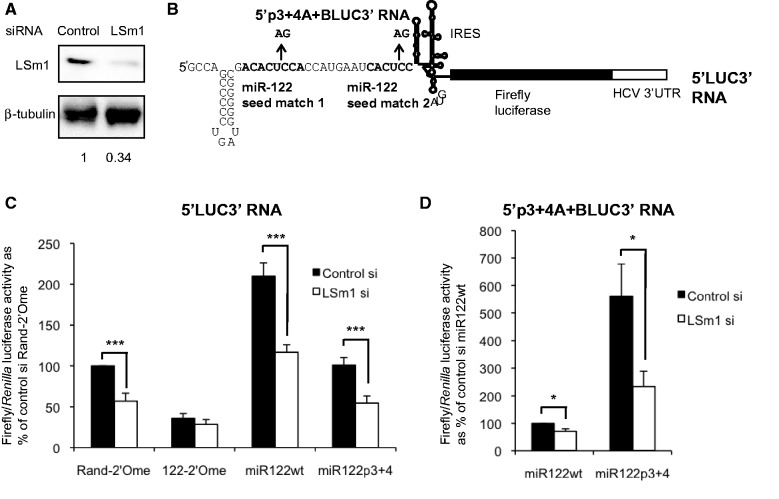
LSm1 contributes to miR-122 activation of HCV IRES-driven translation. (**A**) Western blot showing effective depletion of LSm1 by siRNA transfection in Huh7 cells. LSm1 level relative to β-tubulin is quantified below the image. (**B**) Schematic diagram of the 5′LUC3′ reporter RNA. The miR-122 seed matches in the HCV 5′UTR are mutated at positions 3 and 4 in 5′p3+4A+BLUC3′ RNA. (**C**) Following LSm1 depletion, Huh7 cells were transfected with 5′LUC3′ RNA and a capped polyadenylated *Renilla* luciferase transfection control, in combination with a randomized control 2′O-methylated oligonucleotide (Rand-2′Ome), a 2′O-methylated oligonucleotide that sequesters miR-122 (122-2′Ome), a wild-type miR-122 duplex (miR122wt) or a control duplex with two mutations in the miR-122 seed to abolish target binding (miR122p3+4). LSm1 depletion significantly reduced firefly/*Renilla* luciferase activity in the presence of endogenous miR-122 (Rand-2′Ome, miR122p3+4) or following miR-122 overexpression (miR122wt), but did not significantly affect luciferase production when miR-122 was inhibited (122-2′Ome). (**D**) As (C), except that 5′p3+4A+BLUC3′ RNA was used in place of 5′LUC3′ RNA. The RNA was delivered into cells with the miR122wt duplex, which does not bind to the mutant target sites, or miR122p3+4, which binds and activates translation. Basal luciferase activity in the presence of miR122wt decreased when LSm1 was depleted, but activation by miR122p3+4 decreased further. All data are the mean of at least three independent experiments, +SD. *P < 0.05, ***P < 0.0005.

We first established that we could effectively deplete LSm1 in Huh7 cells by siRNA transfection (Figure 1A). This led to a 40–50% decrease in HCV RNA in cells electroporated with the replication-competent genotype 1a H77ΔE1/p7 RNA (37) (Supplementary Figure S1B and C), confirming that LSm1 contributes to the accumulation of HCV RNA (17,28). We tested the effects of LSm1 depletion on HCV RNA stability by electroporation of H77ΔE1/p7 RNA with either the active site of the NS5B polymerase (H77-AAG) or the miR-122 binding sites mutated (H77-S1+2:p3+4) (36); both mutations abolish HCV replication. Total RNA was harvested 6 h after electroporation. The level of both HCV RNAs was unaffected by LSm1 depletion (Supplementary Figure S1D and E), showing that LSm1 regulates HCV replication without affecting viral RNA stability.

Next, we examined the role for LSm1 in miR-122–mediated regulation of HCV translation by transfection of *in vitro* transcribed reporter RNA in which the firefly luciferase coding region is flanked by the HCV 5′ and 3′ UTRs (5′LUC3′) (Figure 1B). We have previously demonstrated that translation of this RNA is stimulated by miR-122 (11). Following siRNA-mediated depletion of LSm1 in Huh7 cells, 5′LUC3′ reporter RNA was delivered by lipofection with or without sequestration or overexpression of miR-122. Firefly luciferase activity was determined at 6 h after transfection relative to a *Renilla* luciferase transfection control (Figure 1C). In cells containing a randomized control 2′-O-methylated oligonucleotide (Rand-2′Ome) such that endogenous miR-122 function is unaffected, LSm1 depletion led to a 45% reduction in luciferase activity. RNA stability was unaffected (Supplementary Figure S2A), indicating that LSm1 contributes to translation of this reporter RNA.

When an antisense oligonucleotide to inhibit miR-122 was introduced into cells (122-2′Ome), LSm1 depletion resulted in only a 20% decrease in luciferase activity, which was not statistically significant (Figure 1C). When miR-122 was overexpressed (miR122 wt), reporter translation was reduced by 46% when LSm1 was knocked down, similar to the inhibition in the presence of endogenous miR-122. A mutant miR-122 control duplex (miR122p3+4) behaved similarly to the Rand-2′Ome control (Figure 1C). LSm1 depletion therefore leads to a greater inhibition of HCV IRES-driven translation in cells that contain endogenous (Rand-2′Ome, miR122p3+4) or overexpressed miR-122 (miR122wt) than in cells in which miR-122 is sequestered (122-2′Ome). We confirmed that luciferase RNA levels were unaffected by miR-122 depletion or overexpression, as shown previously (11), and did not change when LSm1 was depleted (Supplementary Figure S2A). As measurement of RNA stability following lipofection can give inaccurate results (41), we also determined the level of 5′LUC3′ reporter RNA following electroporation, and confirmed that it is unaffected by miR-122 inhibition or LSm1 depletion (Supplementary Figure S2B). We observed a similar role for LSm1 in miR-122–mediated translation stimulation of a reporter RNA in which the HCV 3′UTR is replaced by a poly(A) tail (Supplementary Figure S3B). These results suggest that LSm1 contributes to miR-122 regulation of translation via the HCV 5′UTR.

We then carried out the same experiment in cells transfected with a reporter RNA in which both miR-122 seed matches are mutated at positions 3 and 4, preventing interaction with wild-type miR-122. This 5′p3+4A+BLUC3′ RNA is bound and activated by mutant miR122p3+4 (11). As endogenous miR-122 does not regulate this reporter, we were able to measure basal translation in the absence of miRNA binding, and found that LSm1 depletion reduced luciferase activity by 30% (miR122wt control transfection, Figure 1D). While this indicates that LSm1 stimulates HCV IRES-driven translation independently of miR-122, we observed a 2-fold greater reduction in luciferase activity on LSm1 knockdown when translation was activated by miR122p3+4 transfection (58%, miR122p3+4, Figure 1D). We obtained similar results with a second mutant reporter RNA in which the miR-122 seed matches were completely replaced with miR-21 seed matches, and a miR21/122 chimera was used to activate translation (Supplementary Figure S3C). In conclusion, wild-type and mutant reporters all show a minor (20–30%) reduction in translation on LSm1 knockdown in the absence of miR-122 regulation, but a 2-fold increase in translation inhibition by LSm1 depletion under conditions of miRNA activation. This leads us to conclude that, while LSm1 has a minor miR-122–independent effect on HCV translation, it also contributes to stimulation of HCV IRES-driven translation by miR-122.

### Regulation of HCV IRES-driven translation by miR-122 is affected differently by different P body proteins

**Figure 2.**
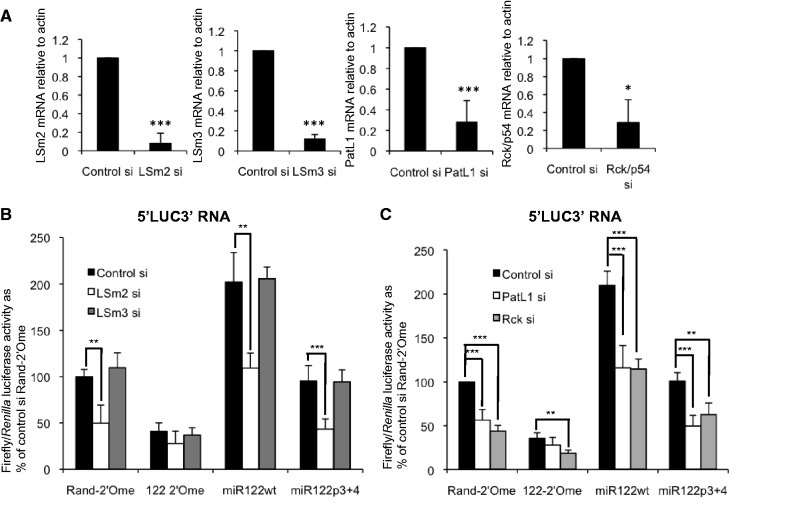
Different P body proteins make different contributions to miR-122–mediated activation of HCV IRES-driven translation. (**A**) qPCR showing effective depletion of LSm2, LSm3, PatL1 and Rck/p54 by transfection of the respective siRNAs. Levels of each mRNA are shown relative to actin mRNA. (**B**) Huh7 cells treated with siRNAs to deplete LSm2 or 3 were transfected with 5′LUC3′ RNA and Rand-2′Ome or 122-2′Ome oligonucleotides, or miR122wt or miR122p3+4 duplexes. LSm2 depletion strongly reduced luciferase activity in the presence of endogenous (Rand-2′Ome, miR122p3+4) or overexpressed (miR122wt) miR-122, with less effect when miR-122 was inhibited (122-2′Ome), whereas LSm3 depletion did not affect reporter translation or its regulation by miR-122. Firefly luciferase activity is shown relative to a *Renilla* luciferase transfection control as a percentage of control siRNA+Rand-2′Ome. (**C**) As (B), except that Huh7 cells were treated with siRNAs to deplete PatL1 or Rck/p54. PatL1 depletion strongly reduced luciferase activity in the presence of endogenous (Rand-2′Ome, miR122p3+4) or overexpressed (miR122wt) miR-122, with less effect when miR-122 was inhibited (122-2′Ome). Rck/p54 knockdown repressed 5′LUC3′ translation to a similar extent irrespective of whether miR-122 was inhibited or overexpressed. All data are an average of at least three independent experiments, +SD. *P < 0.05, **P < 0.005, ***P < 0.0005.

We also examined the effects of depletion of other components of the LSm1–7 complex and P body proteins on translation of the 5′LUC3′ reporter RNA. LSm1 or LSm2 depletion was effective (Figure 2A) and resulted in a decrease in HCV RNA levels in Huh7 cells containing a stable HCV replicon (38), whereas LSm3 depletion did not affect HCV replication (Supplementary Figure S4A). LSm2 depletion also led to similar effects to LSm1 knockdown on 5′LUC3′ RNA translation and its regulation by miR-122, whereas LSm3 depletion had no effect (Figure 2B). This raises the interesting possibility that LSm1 regulates HCV translation as part of an alternative LSm protein complex to the canonical LSm1–7 heptamer.

As PatL1 and Rck/p54 (DDX6) have similar effects to LSm1 on HCV translation and replication (17), we tested whether these host factors cooperate with miR-122 to regulate HCV 5′UTR-driven translation. PatL1 depletion had similar effects to LSm1 or LSm2 knockdown in our reporter assay (Figure 2C). In contrast, when Rck/p54 was depleted we found that inhibition of reporter translation was almost as strong when miR-122 was sequestered (48% inhibition) as in the presence of endogenous miR-122 (56% inhibition) (Figure 2C). This agrees with previous observations that Rck/p54 and miR-122 regulate HCV independently (29), although our results cannot exclude a small cooperative effect of the two factors on HCV translation. Rck/p54 was previously found to stimulate HCV translation in one study, but not another, and a third study found the effects on translation to depend on the passage of Huh7.5 cells used (17,25,29). In our hands, in Huh7 cells, we find that Rck/p54 stimulates HCV IRES-driven translation.

### LSm1 does not affect miR-122–mediated repression at 3′UTR sites or cleavage at a complementary site

**Figure 3.**
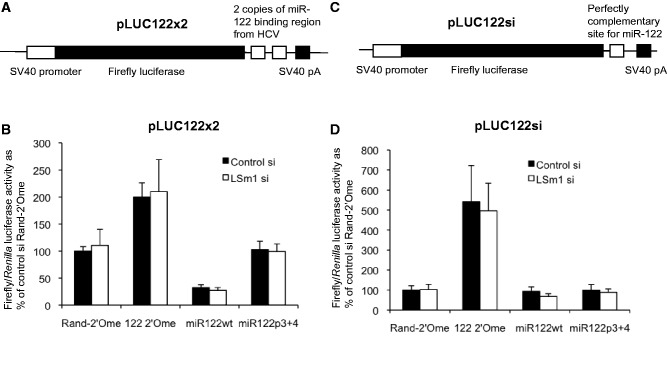
LSm1 does not affect miR-122–mediated repression via 3′UTR sites or RNA cleavage at a perfectly complementary site. (**A**) The pLUC122x2 plasmid, containing two copies of the miR-122 binding region from HCV in the firefly luciferase 3′UTR. (**B**) Huh7 cells treated with control or LSm1 siRNA were transfected with pLUC122x2 together with Rand-2′Ome or 122-2′Ome oligonucleotides, or miR122wt or miR122p3+4 duplexes. The reduction in luciferase activity when miR-122 was overexpressed (miR122wt) or relief of inhibition when miR-122 was sequestered (122-2′Ome) were both unaffected by LSm1 depletion. (**C**) The pLUC122si plasmid, containing a single perfectly complementary site for miR-122 in the 3′UTR. (**D**) pLUC122si was introduced into cells as in (B). Relief of inhibition when miR-122 was sequestered (122-2′Ome) was unaffected by LSm1 depletion. All data are mean firefly luciferase activity relative to a *Renilla* luciferase transfection control in at least three independent experiments, +SD.

To assess whether LSm1 also contributes to the repressive activity of miR-122 binding to 3′UTR sites, we used a luciferase reporter plasmid with two copies of the miR-122 binding region from HCV inserted in the 3′UTR (pLUC122x2, Figure 3A). We have previously shown that miR-122 effectively inhibits translation of the RNA produced from this plasmid (36). Interestingly, we found that basal translation and miR-122 regulation of this 3′UTR reporter were unaffected by LSm1 depletion (Figure 3B). We also examined the effect of LSm1 and miR-122 on luciferase expression from a reporter bearing an exactly complementary miR-122 site, such that miR-122 directs cleavage of the reporter mRNA (pLUC122si, Figure 3C). We found that LSm1 depletion did not affect basal luciferase expression from this plasmid, or the increase in firefly luciferase activity that occurs when miR-122 is inhibited by 122-2′Ome transfection (Figure 3D). Our results demonstrate that both miRNA and siRNA-like repression by miR-122 are unaffected by LSm1. Similarly, we found that depletion of LSm2 or LSm3 did not affect miR-122 repression at 3′UTR sites in either reporter (Supplementary Figure S4B and C). These results imply that LSm proteins play a specific role in miR-122–mediated translation activation via the HCV 5′UTR and not in other functions of miR-122.

### LSm1 does not affect miR-122–RISC recruitment to the HCV 5′UTR

**Figure 4.**
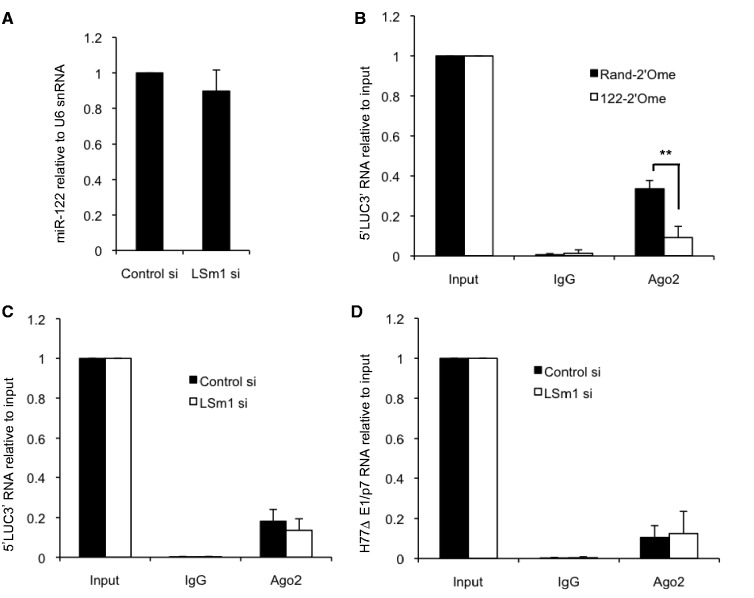
LSm1 does not affect miR-122-RISC association with the HCV 5′UTR. (**A**) miR-122 levels determined by qPCR relative to U6 snRNA in Huh7 cells did not change significantly following LSm1 depletion. (**B**) Huh7 cells were treated with an antisense oligonucleotide to sequester miR-122 (122-2′Ome) or a randomized control (Rand-2′Ome) for 48 h before electroporation with 5′LUC3 RNA. Six hours after electroporation, cell lysates were immunoprecipitated with an anti-Ago2 monoclonal antibody or a normal rat IgG control. Immunoprecipitated RNA levels relative to input were determined by qPCR, and demonstrate that the association of Ago2 with 5′LUC3′ RNA is miR-122-dependent. (**C**) As (B), except that cells were treated with an siRNA specific to LSm1, or a control nontargeting siRNA, instead of the oligonucleotides in (B). LSm1 depletion did not significantly affect Ago2 association with 5′LUC3′ RNA. (**D**) As (C), except that cells were electroporated with H77ΔE1/p7 RNA instead of 5′LUC3′ RNA. All data represent an average of three independent experiments, +SD. **P < 0.005.

Our subsequent investigation focused specifically on the interplay between LSm1 and miR-122 in regulation of HCV. We found that miR-122 expression was unaffected by LSm1 depletion (Figure 4A). Both our group and others have shown that the Argonaute (Ago) proteins function in miR-122–mediated regulation of HCV (11,12). Using a monoclonal antibody specific to Ago2, we were able to coimmunoprecipitate 5′LUC3′ RNA from Huh7 cells 6 h after electroporation. Pretreatment with an antisense oligonucleotide to sequester miR-122 reduced the amount of 5′LUC3′ RNA in the Ago2 immunoprecipitate, indicating that the interaction is mediated through miR-122–RISC recruitment to the RNA (122-2′Ome, Figure 4B). We observed no enrichment of 5′LUC3′ RNA with mutated miR-122 binding sites in the Ago2 immunoprecipitate (data not shown), confirming the specificity of this interaction.

We then carried out Ago2 immunoprecipitation in Huh7 cells electroporated with 5′LUC3′ RNA following siRNA-mediated depletion of LSm1. Of note, the enrichment of 5′LUC3′ RNA in Ago2 immunoprecipitates is reduced in both control and LSm1 siRNA-transfected cells compared to cells that do not contain siRNA (Figure 4B and C). This is likely to be due to competition between transfected siRNA and endogenous miRNA for RISC association. We found that the association of Ago2 with 5′LUC3′ RNA was unaffected by LSm1 knockdown compared with control siRNA treatment (Figure 4C), demonstrating that LSm1 is not required for miR-122–RISC recruitment to 5′LUC3′ RNA. Our results imply that LSm1 regulates miR-122 activation of HCV IRES-driven translation after the miRNA binds. We also carried out this experiment in Huh7 cells 6 h after electroporation with replication-competent H77ΔE1/p7 RNA and observed no effect of LSm1 depletion on the association of Ago2 with HCV RNA (Figure 4D). Together, these results indicate that LSm1 does not affect the binding of miR-122–loaded RISC to the HCV 5′UTR.

### LSm1 is not required for miR-122 to regulate HCV replication

**Figure 5.**
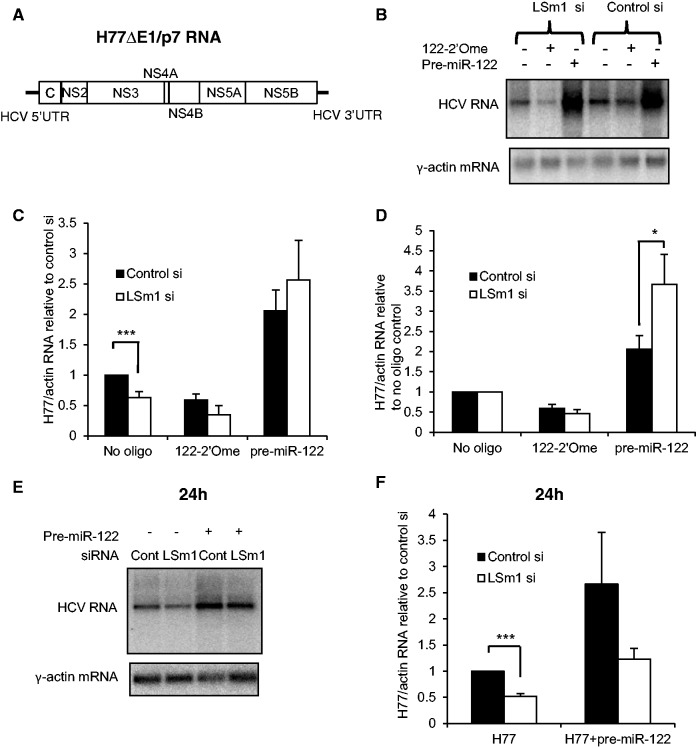
LSm1 is not required for endogenous or overexpressed miR-122 to regulate HCV replication. (**A**) Structure of H77ΔE1/p7 RNA. (**B**) Huh7 cells were electroporated with H77ΔE1/p7 RNA, cultured for 24 h, then transfected with a control siRNA or an siRNA targeting LSm1, with or without cotransfection of 122-2′Ome or synthetic pre-miR-122. HCV and actin RNA were detected by northern blotting. (**C**) As (B), except that HCV RNA levels were determined relative to actin mRNA by qPCR. miR-122 overexpression overcame inhibition of HCV replication by LSm1 knockdown. (**D**) The data shown in (C) replotted relative to the no oligonucleotide control for each siRNA. (**E**) Following LSm1 depletion, Huh7 cells were electroporated with H77ΔE1/p7 RNA, with or without synthetic pre-miR-122. Total RNA was harvested at 24 h after electroporation and analyzed by northern blotting. Pre-miR-122 overexpression increased the level of HCV RNA in both control and LSm1 siRNA-transfected cells. (**F**) As (E), except that HCV RNA levels were determined relative to actin mRNA by qPCR. qPCR data represent an average of three independent experiments, +SD. *P < 0.05, ***P < 0.0005.

Although miR-122 binding to the HCV 5′UTR stimulates HCV IRES-driven translation and/or increases RNA stability, this effect is not sufficient to explain its major role in HCV replication, implying that other stages of the HCV life cycle are also regulated (14,15). We tested whether LSm1 and miR-122 cooperate to regulate HCV replication by electroporating Huh7 cells with H77ΔE1/p7 RNA (Figure 5A) and plating for 24 h before LSm1 or control siRNA transfection. The 122-2′Ome oligonucleotide or pre-miR-122 was included in the transfection to assess the effects of miR-122 sequestration or overexpression. By northern blotting and qPCR, we observed that LSm1 reduced HCV replication but still allowed regulation by miR-122 (Figure 5B and C). The relative decrease in HCV RNA levels on miR-122 inhibition was slightly, although not significantly, greater in LSm1-depleted (53% reduction) than control siRNA-treated (41% reduction) cells (122-2′Ome, Figure 5D). Supplementation with exogenous pre-miR-122 increased HCV RNA levels in LSm1-depleted cells to an even greater extent than in control cells (Figure 5D), such that overexpressed miR-122 overcame the inhibition of HCV replication on LSm1 knockdown (Figure 5C). Together, these results indicate that LSm1 is not required for endogenous or overexpressed miR-122 to regulate HCV replication, in contrast to its effects on translation.

As these experiments were carried out in cells in which HCV replication was established, we also examined whether LSm1 and miR-122 independently regulate early stages of HCV replication. Huh7 cells were treated with control or LSm1 siRNAs for 72 h before electroporation of H77ΔE1/p7 RNA, with or without synthetic pre-miR-122. Total RNA was harvested at 24 h after electroporation, and analyzed by northern blotting (Figure 5E) and qPCR (Figure 5F). Co-electroporation of pre-miR-122 increased HCV RNA levels irrespective of whether LSm1 was knocked down, but did not overcome the effects of LSm1 depletion. The pre-miR-122–dependent increase in HCV RNA was slightly lower in LSm1 siRNA (2.4-fold) than control siRNA-transfected cells (2.7-fold) but was not significantly different.

### LSm1 contributes to activation of infectious HCV translation by miR-122

**Figure 6.**
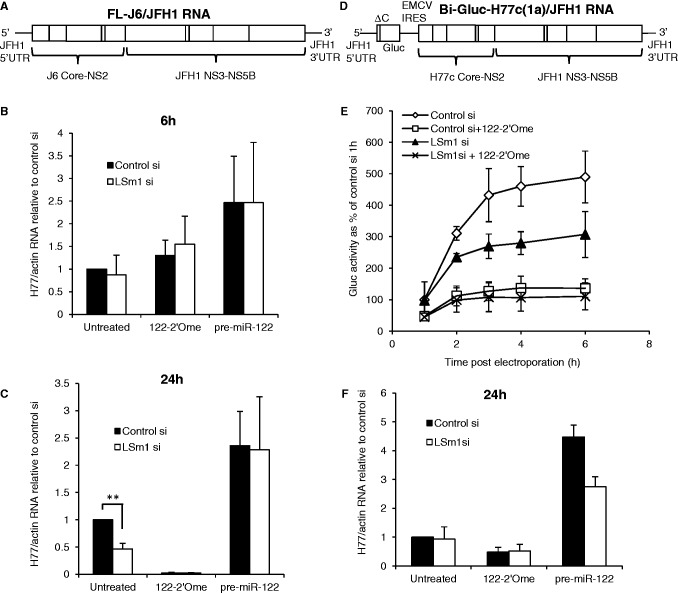
LSm1 contributes to miR-122–mediated regulation of infectious HCV translation. (**A**) Schematic diagram of the infectious FL-J6/JFH1 RNA. (**B**) Following treatment with an siRNA to deplete LSm1 or a nontargeting control, Huh7 cells were electroporated with FL-J6/JFH1 RNA with or without the 122-2′Ome oligonucleotide to sequester miR-122, or synthetic pre-miR-122. Total RNA was harvested at 6 h after electroporation and analyzed by qPCR. HCV RNA relative to actin mRNA was determined relative to control siRNA-treated cells without oligonucleotide. (**C**) As (B), except that RNA was harvested at 24 h after electroporation. Pre-miR-122 overexpression overcame the repressive effects of LSm1 depletion. (**D**) The Bi-Gluc-H77C(1a)/JFH1 infectious RNA, in which *Gaussia* luciferase translation is driven by the JFH1 5′UTR. (**E**) Huh7 cells with or without LSm1 depletion were electroporated with Bi-Gluc-H77C(1a)/JFH1 RNA, with or without 122-2′Ome to sequester miR-122. Cell supernatants harvested over a time course after electroporation were assayed for Gluc activity, which is shown as a percentage of the control siRNA 1 h value. miR-122 inhibition reduced translation to a similar extent with or without LSm1 depletion. (**F**) As (E), except that total RNA was extracted from the cells at 24 h after electroporation and analyzed by qPCR as in (B). LSm1 inhibited replication when pre-miR-122 was overexpressed. All data are an average of at least three independent experiments, +SD. **P < 0.005.

Finally, we examined the effects of LSm1 and miR-122 on translation and replication of infectious HCV RNA. Surprisingly, LSm1 depletion did not significantly affect HCV replication when monocistronic FL-J6/JFH1 RNA was introduced into the Huh7.5 cells that are generally used for infectious virus work (Supplementary Figure S5B), despite similar efficiency of knockdown to Huh7 cells (Supplementary Figure S5C). We therefore carried out these experiments in Huh7 cells. Cells were depleted of LSm1 before electroporation with FL-J6/JFH1 RNA (Figure 6A) (39). LSm1 knockdown did not affect HCV RNA levels at 6 h post electroporation (Figure 6B), but led to a 53% decrease at 24 h. Co-electroporation of 122-2′Ome to sequester miR-122 strongly reduced HCV replication at 24 h; under these conditions, there was no further effect of LSm1 depletion, but as replication was almost abolished we cannot conclude whether endogenous miR-122 and LSm1 cooperate to regulate FL-J6/JFH1 replication. Overexpression of miR-122 stimulated FL-J6/JFH1 replication in both control and LSm1 knockdown cells at 24 h, and reversed the inhibitory effect of LSm1 depletion (pre-miR-122, Figure 6C), in agreement with our observations with H77ΔE1/p7 RNA (Figure 5C).

To examine the effects of miR-122 and LSm1 on infectious HCV translation, we used a bicistronic infectious HCV RNA in which the JFH1 (genotype 2a) 5′UTR drives translation of secreted *Gaussia* luciferase (Gluc), and translation of an H77-JFH1 fusion polyprotein is under the control of the EMCV IRES (Figure 6D). Following knockdown of LSm1 in Huh7 cells, this Bi-Gluc-H77c(1a)/JFH1 RNA was introduced by electroporation with or without miR-122 inhibition or overexpression. HCV RNA levels were unchanged by miR-122 inhibition or LSm1 depletion at 6 h after electroporation (Supplementary Figure S6), indicating that replication had not occurred by this time point and that Gluc was produced by translation of input RNA. LSm1 depletion reduced translation by 25% relative to control siRNA-treated cells at 2 h after electroporation, and by ∼40% at 3–6 h (Figure 6E). Sequestration of miR-122 by 122-2′Ome led to a 50–70% decrease in translation over the time course. There was no significant difference in the translation profile of 122-2′Ome-treated cells with or without LSm1 depletion (Figure 6E), supporting our conclusion that miR-122 and LSm1 cooperate to regulate HCV translation. HCV RNA levels in Huh7 cells electroporated with Bi-Gluc-H77c(1a)/JFH1 RNA at 24 h after electroporation were not significantly affected by LSm1 depletion and only slightly decreased by miR-122 sequestration (Figure 6F). This is likely to be due to the low replication of this RNA in these cells (Supplementary Figure S7A). HCV RNA levels were increased by pre-miR-122, and this increase was reduced by LSm1 depletion (Figure 6F), suggesting that LSm1 may contribute to induction of early replication of this RNA by miR-122 overexpression.

## DISCUSSION

In this study, we identify LSm1 as a cofactor for miR-122 to stimulate HCV IRES-driven translation (Figures 1 and 6). Our results confirm previous observations that LSm1 contributes to HCV replication and translation (17,28), but extend this earlier work by demonstrating that the effect on translation involves miR-122. We note that LSm1 does make a small contribution to translation of luciferase reporters in the absence of miR-122 binding, but we consistently observe at least a 2-fold increase in the response to LSm1 when miR-122 regulation occurs. We observe no effect of either LSm1 or miR-122 on reporter RNA stability (Supplementary Figure S2), indicating that regulation is at the level to translation. In contrast to Scheller *et al.*(17), we find that the HCV 3′UTR is not required for regulation of reporter translation by LSm1 and miR-122 (Supplementary Figure S3B). Differences in experimental techniques, such as the knockdown method, time of transfection or the precise nature of the reporter RNA might account for this disparity, but as regulation at the 5′UTR was the main focus of this investigation, we did not pursue this question.

We find that LSm1 does not contribute to translation repression mediated by miR-122 binding to 3′UTR sites or the siRNA-like activity that occurs when miR-122 encounters a perfectly complementary site (Figure 3). While LSm1–7 and miRNA pathway components colocalize in P bodies, it was previously shown that P body depletion by LSm1 knockdown does not affect miRNA or siRNA-mediated silencing (42), in agreement with our data. The role for LSm1 in regulation of miR-122 activity is therefore specific to activation of translation at the HCV 5′UTR, providing an intriguing first example of a protein factor that is not required for miRNA repressive functions but does contribute to a specific alternative miRNA activity. The mechanisms that allow a miRNA to mediate the different functions of translational repression by binding to 3′UTR sites or activation of translation/viral replication by binding to 5′UTR sites are unclear. Our work provides an insight into these different mechanisms, indicating that specific protein factors may play a role. We previously demonstrated that the core miRISC components Ago1–4 are required for miR-122 to stimulate translation via the HCV 5′UTR, whereas the TNRC6 proteins, which are also essential for miRNA-mediated repression, only play a minor role in this process (11). Together, these results suggest that specialized miRISCs may mediate specific miRNA functions.

We confirm the published observation that LSm1 depletion does not affect the level of miR-122 (28). We also establish that Ago2 is specifically recruited to the HCV 5′UTR by miR-122, in agreement with recent studies (13,43). We find that this miR-122–RISC recruitment is unaffected by LSm1 knockdown (Figure 4), implying that LSm1 regulates miR-122 activity at the HCV 5′UTR subsequent to target binding. These results agree with our observation that LSm1 does not regulate miR-122 regulation at 3′UTR sites (Figure 3), as we would expect these processes to be inhibited by LSm1 depletion if miR-122 levels or target binding by miR-122–RISC were affected. Instead, LSm1 appears to be specifically involved in the activation of HCV IRES-driven translation that occurs following miR-122 binding. While this argues against a chaperone function in target binding by small RNAs similar to that of bacterial Hfq (35), LSm1 may act as a chaperone for secondary or tertiary structural changes in the HCV 5′UTR when miR-122 is bound. Such a role was recently proposed for LSm1, PatL1 and Dhh1 in flock house virus replication in yeast, as these proteins specifically affect the generation of viral RNA3, which depends on long-range RNA–RNA interactions in RNA1 (44). The LSm1–7 complex binds *in vitro* to domain III of the HCV IRES (17), a region that we found was important for miR-122–mediated activation of translation (11). As miR-122 binding causes alterations in HCV 5′UTR structure away from the miR-122 binding sites (16,45), it is possible miR-122 and LSm1 may cooperate to restructure the HCV IRES in a manner that stimulates translation.

In contrast to translation, we find that inhibition of HCV replication by miR-122 sequestration is not affected by LSm1 depletion (Figure 5). This suggests that regulation of HCV translation and replication by miR-122 are different processes with different requirements for host factors such as LSm1. Overexpression of miR-122 can overcome the repressive effects of LSm1 knockdown on HCV replication in this established H77ΔE1/p7 replication system, or at 24 h after electroporation of FL-J6/JFH1 RNA (Figures 5C and 6C). miR-122 overexpression also overcomes the repression of HCV replication induced by depletion of the P body component RCK/p54 (DDX6), which led to the conclusion that miR-122 and Rck/p54 modulate HCV replication by independent mechanisms or possibly by redundant pathways (25).

Importantly, we observed several differences in our translation and replication experiments between the effects of endogenous and overexpressed miR-122, between the different viral RNAs and between Huh7 and Huh7.5 cells. Overexpression of miR-122 in Huh7 cells stimulates 5′LUC3′ reporter translation to the same extent whether or not LSm1 is depleted (Figure 1C). In contrast, both activation of wild-type reporter or viral translation by endogenous miR-122, and activation of mutant reporter RNA translation by mutant miR-122, are more sensitive to LSm1 depletion than basal translation in the absence of miRNA regulation (Figure 1C and D). These results suggest that LSm1 is important for miR-122–mediated stimulation of HCV IRES-driven translation from a low, basal level but does not contribute to further stimulation when miR-122 is increased beyond its endogenous level.

We observed different effects of LSm1 and miR-122 on the three different viral RNAs we tested, which might be due to differences in replication efficiency. At 24 h after electroporation, FL-J6/JFH1 replication is strong (Supplementary Figure S7A) and pre-miR-122 overexpression overcomes the repressive effects of LSm1 depletion (Figure 6C). This agrees with our observations in the context of established H77ΔE1/p7 replication (Figure 5C). In contrast, H77ΔE1/p7 RNA replication at 24 h after electroporation is only 10% of that of FL-J6/JFH1 (Supplementary Figure S7A); pre-miR-122 overexpression stimulates replication when LSm1 is knocked down, but does not overcome the requirement for LSm1 (Figure 5F). We observe no reduction in Bi-Gluc-H77C(1a)/JFH1 RNA levels at 24 h after electroporation on LSm1 depletion (Figure 6F), probably because replication of this RNA is so low that HCV RNA expression is largely a measure of RNA stability (Supplementary Figure S7A). However, LSm1 is required for the replication that occurs following overexpression of pre-miR-122 (Figure 6F). Together, these observations suggest that when HCV replication is efficient and is maximized by miR-122 overexpression, it does not require LSm1. LSm1 is more important for inefficient HCV replication, and perhaps cooperates with miR-122 to contribute to the switch from translation to early replication.

In agreement with this model, we find that LSm1 does not contribute to replication of FL-J6/JFH1 RNA in Huh7.5 cells (Supplementary Figure S5B), where replication is 3-fold more efficient than in Huh7 cells and miR-122 levels are higher (Supplementary Figure S7). LSm1 knockdown is equally efficient in Huh7.5 and Huh7 cells (Supplementary Figure 5C). While other groups have observed stronger effects of LSm1 depletion on JFH1 replication in Huh7.5 cells, our different results may be due to differences between the JFH1-derived RNA or Huh7.5 isolates used, or to less efficient LSm1 knockdown. Translation of 5′LUC3′ or Bi-Gluc-H77C(1a)/JFH1 RNA is also less sensitive to LSm1 depletion in Huh7.5 cells than Huh7 (data not shown). Different Huh7 isolates have been shown to differ in physiology and HCV infectivity (46), while Rck/p54 depletion has different effects on HCV translation in different passages of Huh7.5 cells and in different studies (17,25,29). Together, these observations support the idea that HCV may have different sensitivity to cofactors in different Huh7 or Huh7.5 cell isolates.

HCV infection leads to the disruption of P bodies and relocalization of specific P body proteins to sites of viral replication, while P body disruption by depletion of Dcp2 or TNRC6A does not affect HCV replication. Ago2 and miR-122 also move out of P bodies and associate with lipid droplets in HCV-infected cells (17,26,28,47). Together, these observations suggest that both LSm1 and miR-122 regulate HCV translation and replication outside the P bodies. It is still not clear how P body proteins assist HCV replication, although both our data and that of other groups suggest that at least some of this effect occurs by stimulation of viral translation (Figures 1 and 6) (17). P body proteins including LSm1 have also been implicated in the replication of a number of different RNA viruses (23,48), suggesting that similar mechanisms of regulation may be involved. The P body proteins may contribute to the switch from translation to replication of viral RNA, perhaps by modulating RNA structure or localization of RNA to replication complexes. As P bodies are dynamic, it will be interesting to determine whether viral RNA initially interacts with these regulatory proteins within the P bodies, leading to disruption of these foci, or associates with free pools of the proteins in the cytoplasm and prevents P body formation.

The function of LSm1 in mRNA decapping and decay is not yet fully understood, but several lines of evidence indicate that it has additional functions in RNA metabolism. LSm1–7 binds and stabilizes RNA molecules with 5′ poly(A) tracts, such as the orthopoxvirus mRNAs (49). LSm1 and LSm4 are found in mRNP complexes in neuronal dendrites, where it was proposed that these proteins function in regulation of localized protein synthesis (50). We find that only a subset of the LSm proteins we tested affect HCV IRES-driven translation, raising the possibility that alternative complexes of LSm proteins may exist in cells and regulate viral replication. In the heteroheptameric LSm1–7 ring, LSm1 interacts directly with LSm2 but not LSm3. We observe that LSm1 and LSm2 have similar effects on miR-122 regulation of HCV IRES-driven translation and on HCV replication, whereas LSm3 does not affect this process (Figure 2 and Supplementary Figure S4). Alternative LSm multimers can assemble *in vitro* (51). Our results suggest that LSm1 may function in a complex other than LSm1–7 to stimulate HCV IRES-driven translation in a manner that involves miR-122.

It has been clearly shown that the minor effects of miR-122 on HCV translation or RNA stability are not sufficient to explain the requirement for this miRNA for HCV replication (14,15,16), implying that miR-122 also regulates a later stage of the viral replication cycle. This second regulation event has proven difficult to identify; miR-122 does not affect HCV RNA synthesis either in purified replication complexes, or by 4-thio-uridine labeling of nascent RNA (8,9). It remains possible that miR-122 is important for processes such as initiation of HCV replication. Our observation that LSm1 contributes to miR-122–mediated activation of HCV IRES-driven translation, but is not required for miR-122 to regulate HCV replication, supports the hypothesis that the miRNA exerts multiple effects on the HCV replication cycle.

This work provides new insight into the mechanisms by which both miR-122 and LSm1 regulate HCV, and forms a basis for future research to elucidate the function of these important host factors in more detail.

## SUPPLEMENTARY DATA

Supplementary Data are available at NAR Online.

## FUNDING

Biotechnology and Biological Sciences Research Council David Phillips Fellowship [BB/F02360X/1 to C.L.J.]. Funding for open access charge: University of Nottingham fund.

*Conflict of interest statement*. None declared.

## Supplementary Material

Supplementary Data
