# Supplementary material for: The P body protein LSm1 contributes to stimulation of hepatitis C virus translation, but not replication, by microRNA-122
Source: Nucleic Acids Res. 2013 Oct 18;42(2):1257–69. doi: 10.1093/nar/gkt941 (PMC3902931; doi:10.1093/nar/gkt941)
Supplement: Supplementary Data [file supp_42_2_1257__index.html]

The P body protein LSm1 contributes to stimulation of hepatitis C virus translation, but not replication, by microRNA-122 — The P body protein LSm1 contributes to stimulation of hepatitis C virus translation, but not replication, by microRNA-122 — Supplementary Data 

# The P body protein LSm1 contributes to stimulation of hepatitis C virus translation, but not replication, by microRNA-122

## Supplementary Data

files

**Files in this Data Supplement:**

- Supplementary Data - pdf file
