# Supplementary material for: The P body protein LSm1 contributes to stimulation of hepatitis C virus translation, but not replication, by microRNA-122
Source: Nucleic Acids Res. 2013 Oct 18;42(2):1257–69. doi: 10.1093/nar/gkt941 (PMC3902931; doi:10.1093/nar/gkt941)
Supplement: Supplementary Data [file supp_gkt941_nar-01182-v-2013-File008.pdf]

Target gene	siRNA sense strand or catalogue number
LSm1	GUGACAUCCUGCCACCUCACUU
LSm2	AGAAAUACCCUCACAUGUUUU
LSm3	UAAAUCAACGAAACGGAAUUU
PatL1	ON-TARGETplus SMARTpool L-015591-01
Rck/p54	GCAGAAACCCUAUGAGAUUUU
Control	ON-TARGETplus nontargeting siRNA#3

Supplementary Table 1 siRNA sequences or catalogue numbers. All siRNAs were purchased from Dharmacon.

Primer name	Sequence
HCV qF	CTTCACGCAGAAAGCGTCTA
HCV qR	CAAGCACCCTATCAGGCAGT
JFH1 qF	CTTCACGCAGAAAGCGCCTA
JFH1 qR	CAAGCGCCCTATCAGGCAGT
Actin qF	AGCACAGAGCCTCGCCTTT
Actin qR	TCATCATCCATGGTGAGCTG
Renilla qF	AACGCGGCCTCTTCTTATTT
Renilla qR	GTCTGGTATAATACACCGCG
LSm1 qF	TGTGGAGCGTATTCATGTGGGC
LSm1 qR	AGCTTGGTCTGCTGTTCCACCC
LSm2 qF	ACCTGAGCATCTGTGGAACCCTCC
LSm2 qR	ATCGGACCACTGAGCCCCGA
LSm3 qF	TCACGTTTCCGGAGATTGACGTTGC
LSm3 qR	GGCTGAGCCTGATAAGATCCAGGGG
PatL1 qF	CACCAGCACTCTCCAATCCTCACCT
PatL1 qR	CGCAGAAGTTCTCGTGTTGCCATGA
Rck qF	GGTGCCTACCTCATTCCCTTAC
Rck qR	ATGTGTTTGCTGACCTGGATG

Supplementary Table 2. Primers used for qPCR.

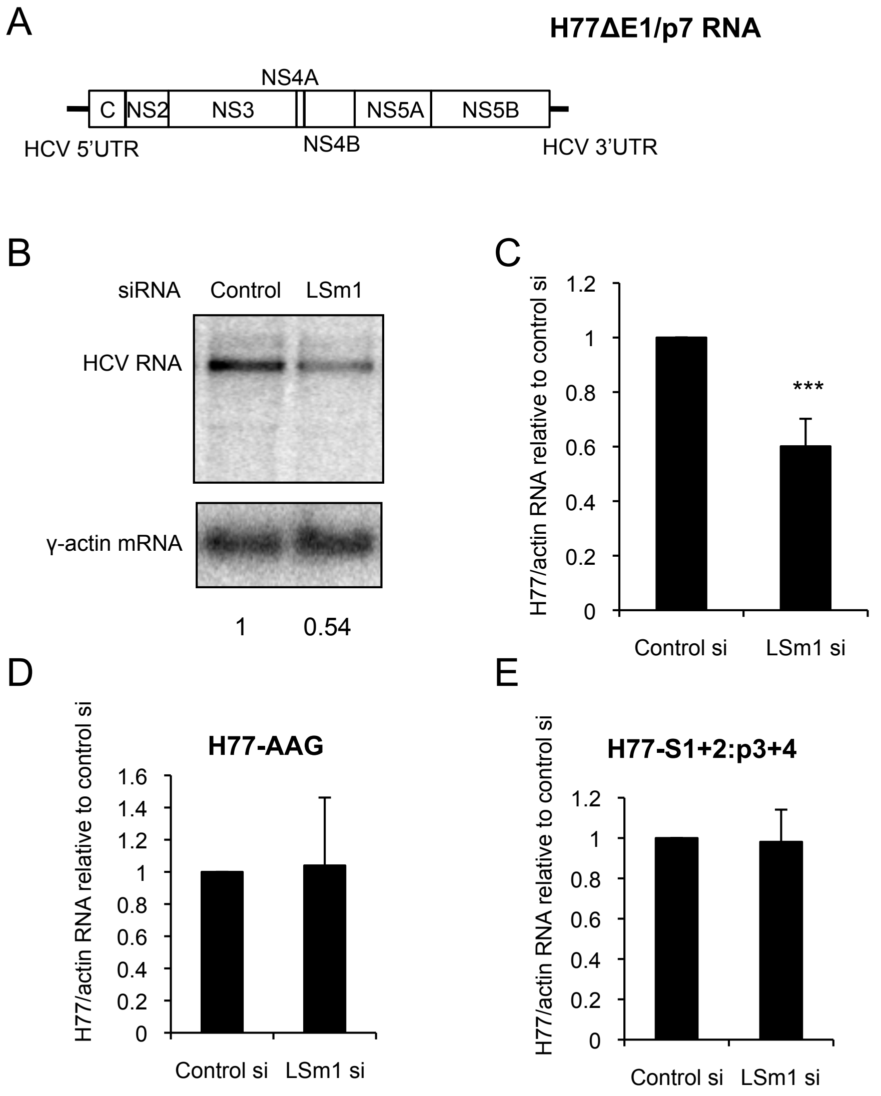

Supplementary Figure 1.LSm1 stimulates replication of H77ΔE1/p7 RNA.(A) Schematic representation of replication-competent H77ΔE1/p7 RNA. (B) Huh7 cells were electroporated with H77ΔE1/p7 RNA before treatment with an siRNA targeting LSm1 or a non-targeting control. A decrease in HCV RNA following LSm1 knockdown was observed by northern blotting. Relative HCV/actin mRNA ratios are quantified below the image. (C) As (B), except that qPCR was used to quantify the decrease in HCV RNA. (D) LSm1 was depleted from Huh7 cells before electroporation with replication-deficient H77-AAG RNA. HCV RNA levels were determined by qPCR relative to an actin mRNA control at 6h post electroporation, and were unaffected by LSm1 knockdown. (E) As (D), except that H77-S1+2:p3+4 RNA, in which both miR-122 binding sites are mutated, was used. LSm1 depletion did not affect stability of this RNA. HCV RNA levels relative to actin mRNA relative to control siRNA transfection are shown as an average of three independent experiments, +SD. *** $P<0.0005$.

A

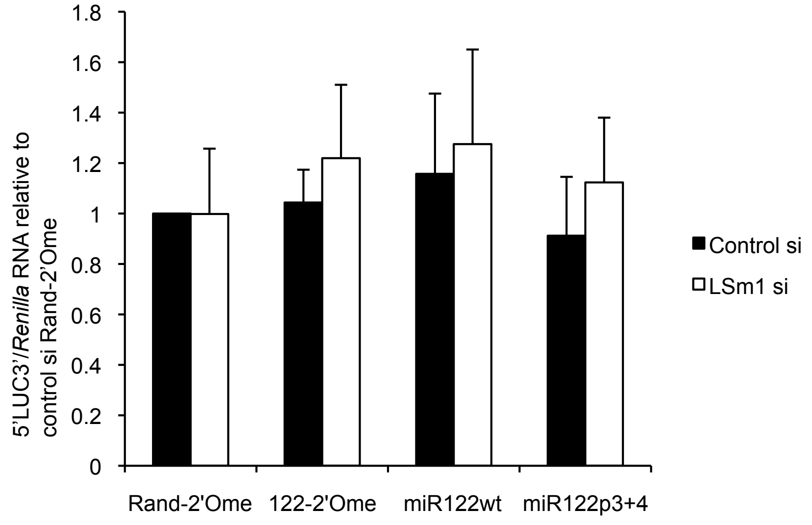

B

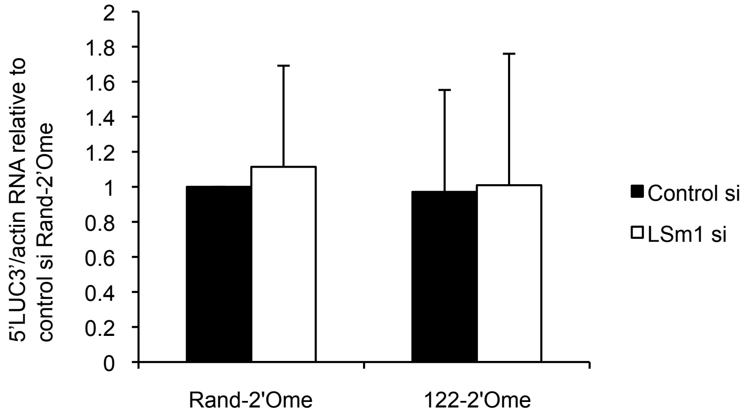

Supplementary Figure 2. 5'LUC3' RNA stability is unaffected by miR-122 or LSm1. (A) Huh7 cells treated with an siRNA specific to LSm1 or a non-targeting control siRNA were transfected with 5'LUC3' RNA. A randomized control oligonucleotide (Rand-2'Ome), an oligonucleotide to sequester miR-122 (122-2'Ome), a wildtype miR-122 duplex (miR122wt) or a mutant control duplex (miR122p3+4) was included in the transfection. The level of 5'LUC3' RNA was determined at 6h post transfection by qPCR relative to a *Renilla* luciferase transfection control RNA. (B) Huh7 cells were treated with an antisense oligonucleotide to sequester miR-122 (122-2'Ome) or a randomized control (Rand-2'Ome) in combination with control or LSm1 siRNA for 48h before electroporation with 5'LUC3' RNA. Total RNA was harvested at 6h post electroporation, and the level of 5'LUC3' RNA determined by qPCR relative to actin mRNA. Data represent the mean of at least three independent experiments, + SD.

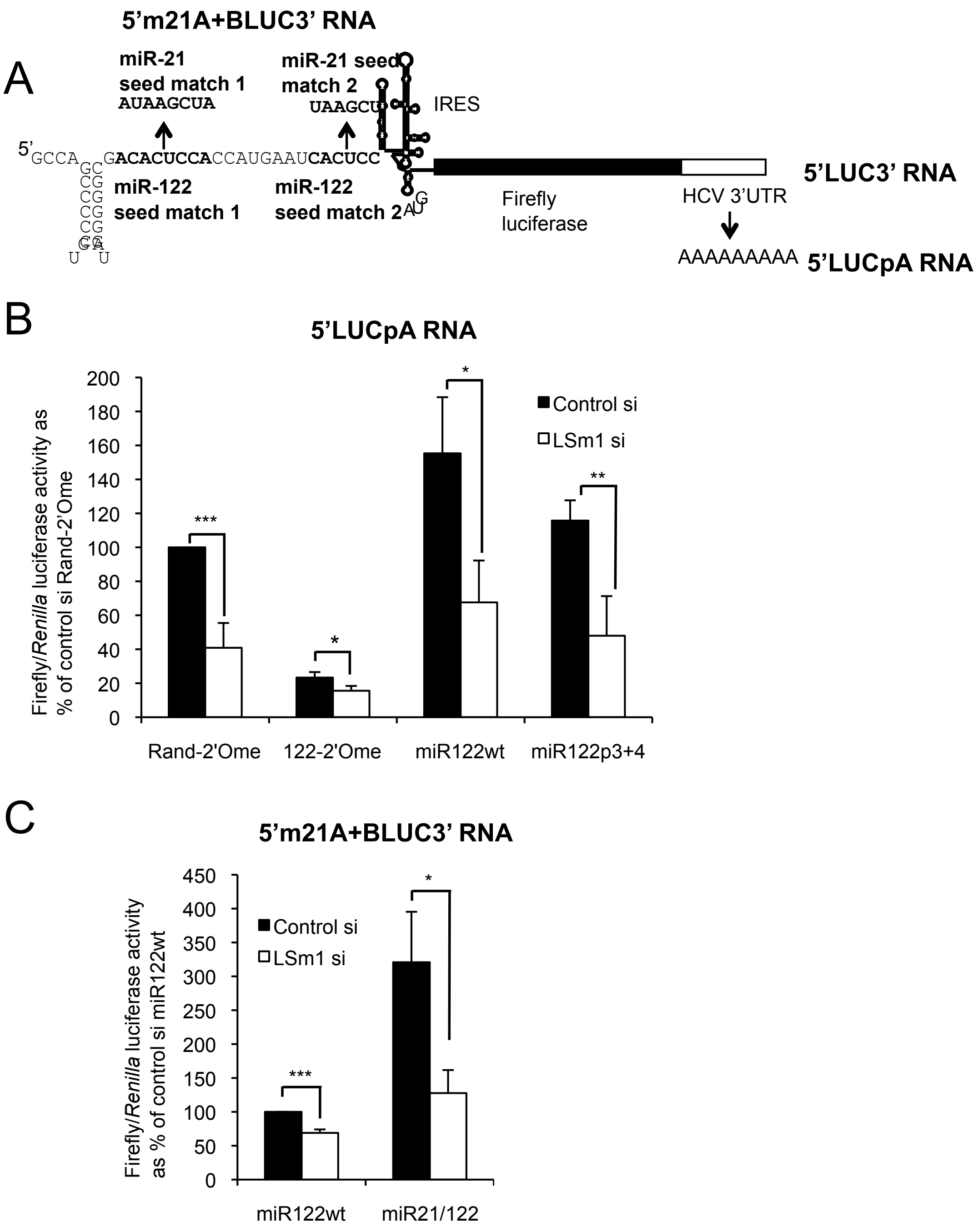

Supplementary Figure 3. The HCV 3'UTR and intact miR-122 binding sites are not required for LSm1 to regulate miR-122 activation of HCV IRES-driven translation. (A) Diagram of the 5'LUC3' reporter, with the HCV 3'UTR replaced with a poly(A) tail to make 5'LUCpA RNA or miR-122 seed matches replaced by miR-21 seed matches in 5'm21A+BLUC3' RNA. (B) Huh7 cells treated with an siRNA to deplete LSm1, or a non-targeting control siRNA, were transfected with 5'LUCpA RNA with miR-122 inhibition or overexpression. LSm1 depletion strongly reduces luciferase activity in the presence of endogenous miR-122 (Rand-2'Ome, miR122p3+4) or following miR-122 overexpression, but has only a minor effect on luciferase production when miR-122 is inhibited (122-2'Ome). (C) As (B), except that 5'm21A+BLUC3' RNA was used. The RNA was delivered into cells with the miR122wt duplex, which does not bind to the mutant target sites, or a chimeric duplex RNA in which the miR-21 seed is fused to the miR-122 3' end, allowing activation of translation (miR21/122). Basal luciferase activity in the presence of miR122wt decreased when LSm1 was depleted, but activation by miR21/122 decreased further. Firefly/*Renilla* luciferase data are an average of three independent experiments, +SD.

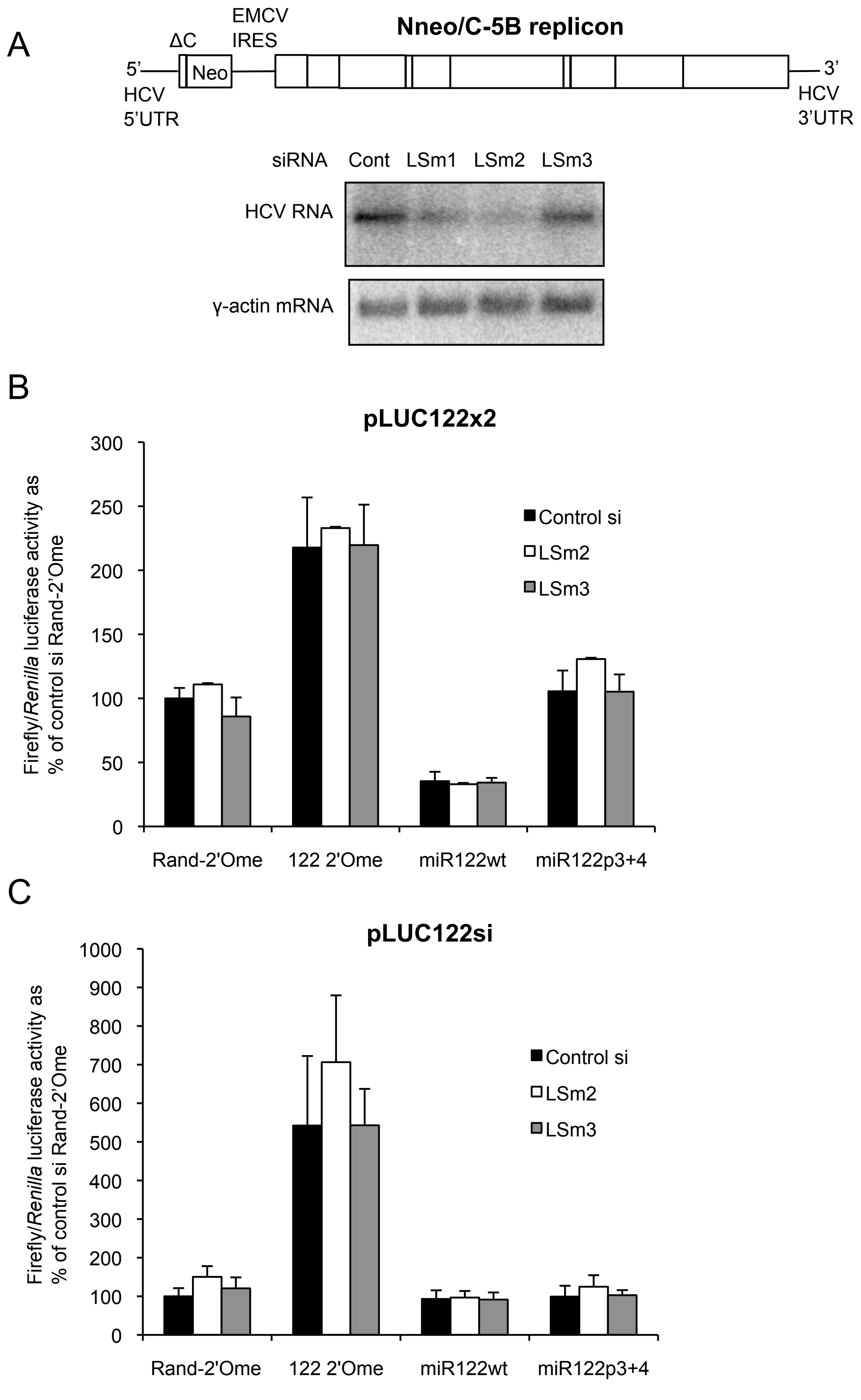

Supplementary Figure 4. LSm2 contributes to HCV replication but does not affect miR-122-mediated repression at 3'UTR sites or cleavage at a complementary site (A) Huh7 cells containing a stable NNeo/C-5B replicon were transfected with siRNAs to deplete LSm1, 2 or 3. HCV RNA levels were determined by northern blotting, and decreased on LSm1 or 2 but not LSm3 depletion. (B) Huh7 cells treated with siRNAs to deplete LSm2 or 3 were transfected with pLUC122x2, containing two copies of the miR-122 binding region from HCV RNA in the luciferase 3'UTR, with inhibition or overexpression of miR-122. Translation repression by miR-122 was unaffected by LSm2 or 3 knockdown. (C) As (B), except that pLUC122si, containing a perfectly complementary target site for miR-122 in the 3'UTR, was used. All data are an average of at least three independent experiments, +SD.

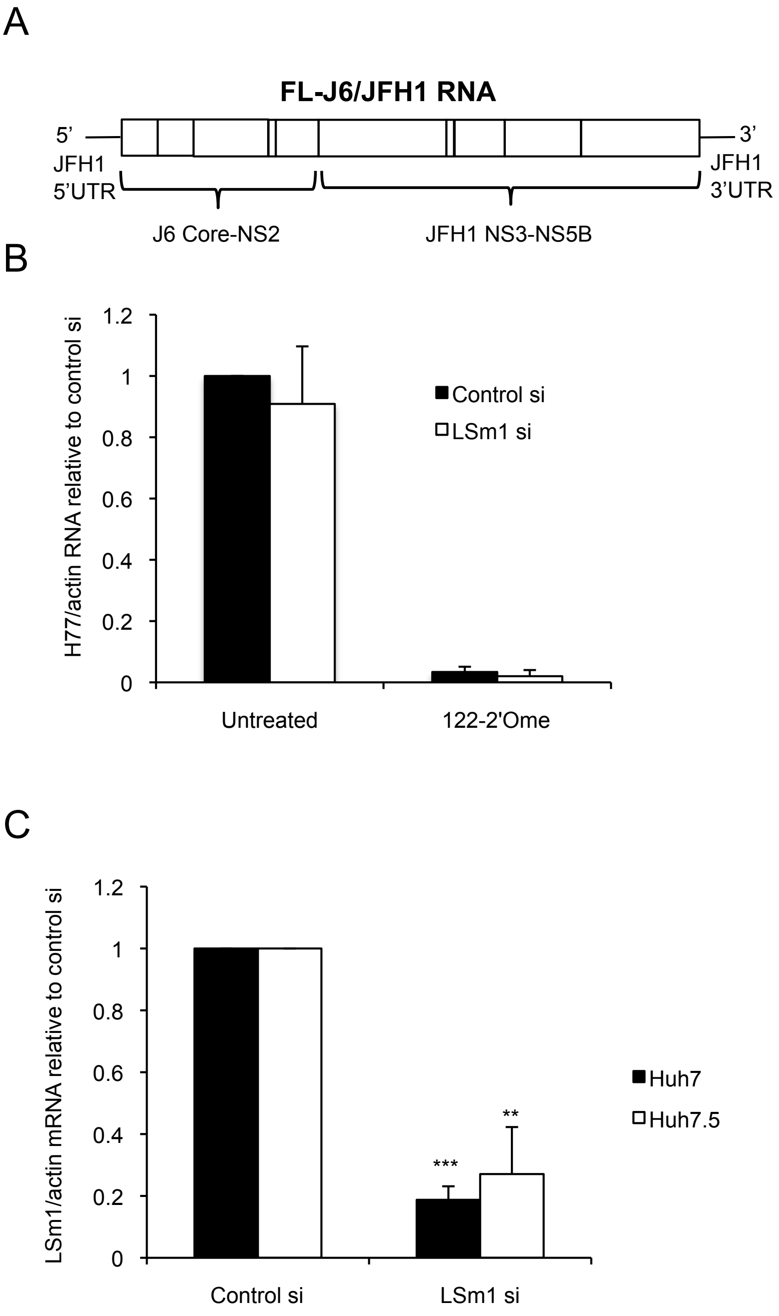

Supplementary Figure 5. LSm1 depletion does not significantly affect replication of monocistronic J6/JFH1 RNA or its regulation by miR-122 in Huh7.5 cells. (A) Diagram showing the FL-J6/JFH1 infectious RNA. (B) Following treatment with an siRNA to deplete LSm1 or a non-targeting control, Huh7.5 cells were electroporated with infectious J6/JFH1 RNA with or without the 122-2'Ome oligonucleotide to sequester miR-122. Total RNA was harvested at 24h post electroporation and analyzed by qPCR. HCV RNA levels relative to actin mRNA are shown relative to control siRNA-treated cells without oligonucleotide. (C) qPCR for LSm1 mRNA relative to actin mRNA showing effective siRNA-mediated knockdown of LSm1 in both Huh7 and Huh7.5 cells. All data are an average of at least three independent experiments, +SD. $^{**}P<0.005$, $^{***}P<0.0005$.

A

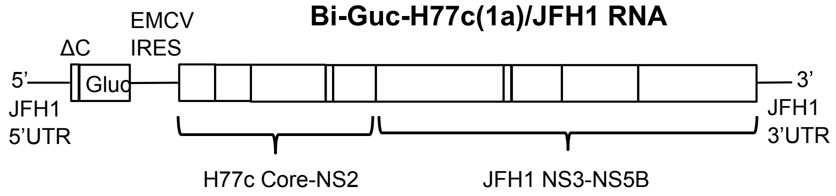

B

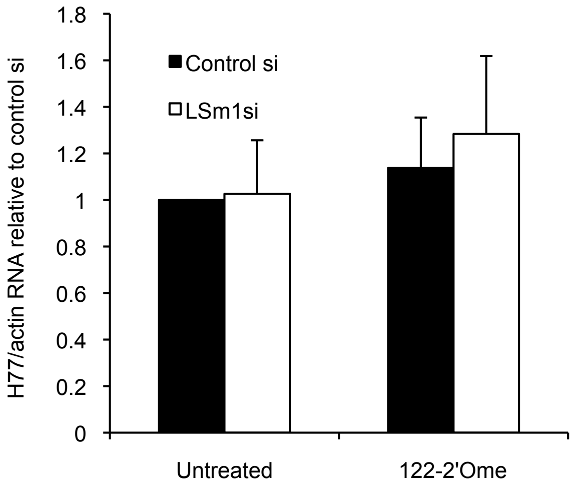

Supplementary Figure 6. Bi-Guc-H77c(1a)/JFH1 RNA levels are unaffected by LSm1 or miR-122 knockdown at 6h post electroporation. (A) Diagram showing the Bi-Guc-H77c(1a)/JFH1 infectious RNA. (B) Huh7 cells with or without LSm1 depletion were electroporated with Bi-Gluc-H77C(1a)/JFH1 RNA, with or without 122-2'Ome to sequester miR-122. Total RNA was harvested at 6h post electroporation and analysed by qPCR. HCV RNA levels relative to actin mRNA were determined relative to control siRNA-treated cells without oligonucleotide. All data are an average of at least three independent experiments, +SD.

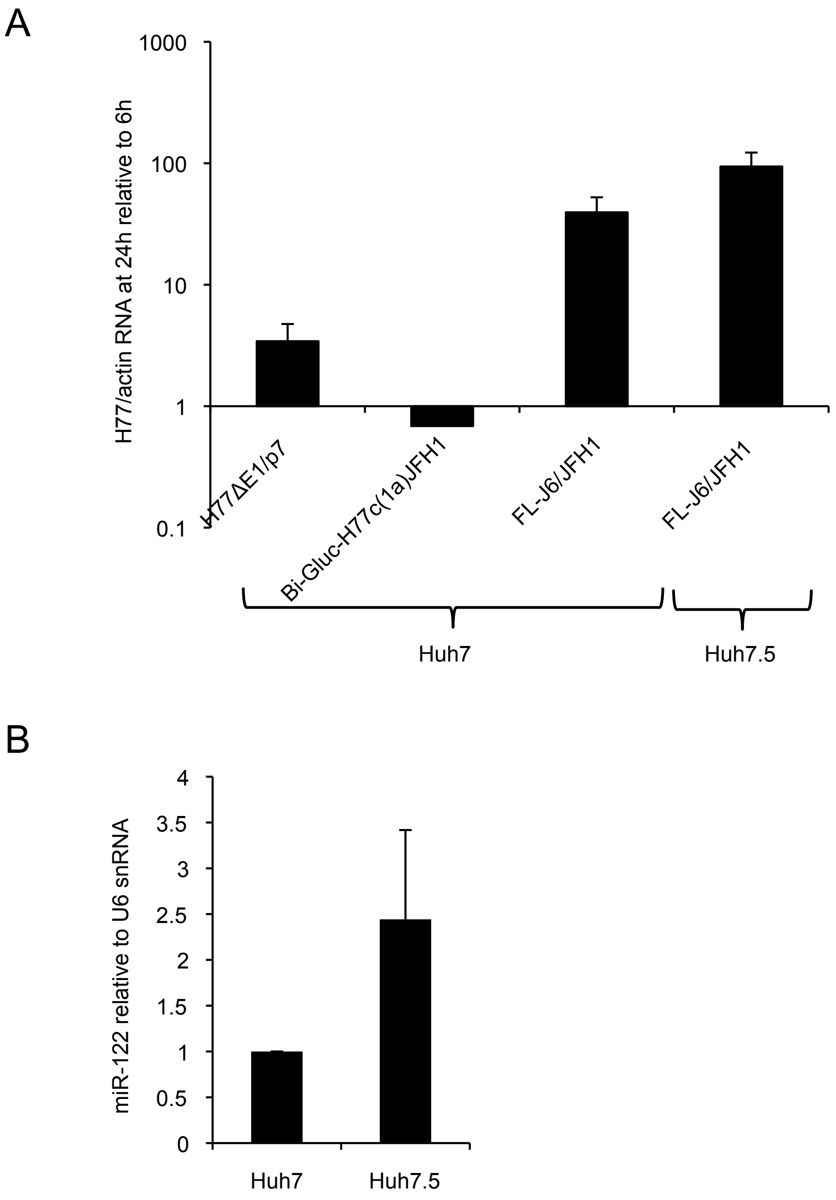

Supplementary Figure 7. Different HCV RNAs replicate with different efficiency. (A) H77ΔE1/p7, Bi-Guc-H77c(1a)/JFH1 and FL-J6/JFH1 RNAs were introduced into Huh7 cells and FL-J6/JFH1 RNA into Huh7.5 cells by electroporation. HCV RNA relative to an actin mRNA control is shown for each RNA at 24h post electroporation compared to 6,h on a logarithmic scale. (B) miR-122 expression relative to U6 snRNA was quantified in Huh7.5 cells relative to Huh7 cells, and is increased. qPCR data are average values from at least three independent experiments, +SD.
